# The Effectiveness of Somatization in Communicating Distress in Korean and American Cultural Contexts

**DOI:** 10.3389/fpsyg.2016.00383

**Published:** 2016-03-23

**Authors:** Eunsoo Choi, Yulia Chentsova-Dutton, W. Gerrod Parrott

**Affiliations:** ^1^Japanese Society for the Promotion Fellowship, Kokoro Research Center, Kyoto UniversityKyoto, Japan; ^2^Department of Psychology, Georgetown University, WashingtonDC, USA

**Keywords:** culture, distress, emotions, somatization, communication, empathy

## Abstract

Previous research has documented that Asians tend to somatize negative experiences to a greater degree than Westerners. It is posited that somatization may be a more functional communication strategy in Korean than American context. We examined the effects of somatization in communications of distress among participants from the US and Korea. We predicted that the communicative benefits of somatic words used in distress narratives would depend on the cultural contexts. In Study 1, we found that Korean participants used more somatic words to communicate distress than US participants. Among Korean participants, but not US participants, use of somatic words predicted perceived effectiveness of the communication and expectations of positive reactions (e.g., empathy) from others. In Study 2, we found that when presented with distress narratives of others, Koreans (but not Americans) showed more sympathy in response to narratives using somatic words than narratives using emotional words. These findings suggest that cultural differences in use of somatization may reflect differential effectiveness of somatization in communicating distress across cultural contexts.

## Introduction

People, especially when distressed, engage in emotional communication to elicit care and support (Schachter, 1959; Bowlby, 1969). Just as infants and children seek to be comforted by their parents or other attachment figures (Bowlby, 1969; Ainsworth et al., 1978), adults are motivated to seek emotional support or comfort from their attachment figures (e.g., romantic partner, spouse, friend) when faced with stressful life events (Shaver and Klinnert, 1982; Rimé, 2009). According to studies of social sharing of emotions, the primary motivation for discussing negative emotions is distress relief, expectation of help, comfort, cognitive clarity, and consolation (Reis and Patrick, 1996; Zech, 2000; Rimé, 2009). Such expectations of understanding, empathy, and support from interaction with partners are not groundless. It has been well documented that being exposed to the negative emotional experience of others evokes vicarious emotional responses in observers (Eisenberg et al., 1991; Preston and de Waal, 2002) and leads to their empathic concern (Davis, 1983).

Naturally, cultural contexts of the emotional exchanges influence the ways in which emotions are expressed and interpreted (Mesquita and Albert, 2007). The basic premise of effective emotional communication is that individuals engage in emotional sharing that is meaningful and appropriate in a given cultural context. Researchers have suggested that cultural scripts help individuals respond effectively to the incentives and constraints in solving problems in their environment (Yamagishi et al., 2008; Yamagishi and Suzuki, 2009; Chiu et al., 2010). Cultural scripts of emotional communication are knowledge structures that are salient to members of a given culture and serve as prompts for automatic and fast retrieval of networks of information (D’Andrade, 1984; Wierzbicka, 1999). In other words, cultural scripts function like conventionalized solutions that are widely accepted and considered effective in the community.

The present work is based on the assumption that the ways in which people communicate emotions in interpersonal contexts may vary depending on the culturally shared meaning systems. The aim of the present work is to show that emotional communication, particularly distress communication, occurs in different ways that have divergent interpersonal outcomes depending on cultural contexts. The focus of this study will be on somatization, one of the best-known examples of cross-cultural variation in emotional communication between the East and the West. Previous research has focused primarily on somatization in relation to intrapersonal cultural differences, such as in mind–body holism, language use, and perception of bodily sensations (see Ryder et al., 2002, for a review). Going beyond the previous literature, the present study shifts the focus to somatization in interpersonal context, that is, as a communication strategy. Specifically, by comparing distress disclosures between participants from Korea and the US, we show that the interpersonal outcomes in the distress disclosures are associated with culturally patterned emotional communication strategies.

Based on the perspective of some researchers who view scripts as socially effective strategies that are likely to have utility (Yamagishi et al., 2008; Chiu et al., 2010), the present study tests the notion that employing somatization is a functional communication strategy that leads to positive interpersonal consequences in cultural contexts that foster somatization.

## Somatization as Interpersonal Communication in Cultural Context

One clear cultural difference in emotional communication can be observed in communication of distress. Research on culture and psychopathology has documented that individuals in non-Western cultural contexts, such as China, experience or express emotional distress with somatic symptoms more frequently than do individuals in North American cultural contexts, such as Canada (Ryder et al., 2002). Most extensive research on somatization was conducted with Chinese people both in China and in North America. Chinese somatization is now a key finding of research on culture and psychopathology (Ryder et al., 2011, 2012). The attention of researchers was first drawn to the somatization of depression due to the extremely low rates of depression in China based on data from systematic large-scale epidemiological surveys. According to Zhang and colleagues (as cited in Ryder et al., 2002), a mental health survey that was undertaken in seven regions of China in 1993 revealed significantly lower rates of depressive disorders compared to those observed in the US. Of the 19,223 people surveyed in 1993, only 16 reported a lifetime depression, which was several 100 times lower than prevalence rates observed in North America. More recently, in the World Health Organization World Mental Health Survey Initiative study that was conducted in Shenzhen, China between 2006 and 2007, depression prevalence rate among Chinese was estimated as 6.5% for lifetime prevalence and 3.8% for 12-month prevalence. Although the prevalence rates in China have increased dramatically in recent decades, they were still significantly lower than those of the US, which were 19.2% for lifetime prevalence and 8.3% for 12-month prevalence (Bromet et al., 2011). The low prevalence of depression is thought to be caused in part by the fact that Western criteria of major depression do not match Chinese scripts for communicating distress.

Against the background of drastically low rates of major depression, neurasthenia (literally meaning “weakness of nerves”) is relatively common among the Chinese. Adopted after the 1949, Cultural Revolution from Russian psychiatrists, the diagnostic category of neurasthenia was translated as *shenjing shuairuo* (SJSI). This term refers to the wide range of symptoms including primarily somatic (e.g., fatigue, or dizziness), cognitive (e.g., poor memory or unpleasant thoughts), and emotional symptoms (e.g., vexatiousness, nervousness). Although emotional symptoms (e.g., depressed mood, pessimism) are present in the symptoms of SJSI, they are not as prominent among the diagnostic criteria (Parker et al., 2001; Kleinman, 1982). Similar to neurasthenia in China, *Hwa-byung* in Korea is an example of another cultural variant in emotional communication of distress. The term hwa-byung is made up of two words: hwa meaning “fire” or “anger” and byung meaning “illness” and it literally means an “illness of anger,” and was recognized as a culture-bound syndrome in the fourth edition of the Diagnostic and Statistical Manual of Mental Disorders (Diagnostic and Statistical Manual of Mental Disorders [DSM-IV], 1994). Hwa-byung also highlights somatic complaints as its primary symptoms. The commonly reported symptoms by patients suffering from hwa-byung are respiratory stuffiness, shortness of breath, headaches, heat-sensations in the face and body, lump in the throat and chest, as well as psychological symptoms including depressed mood and anxiety (Min et al., 2009). There is a shared cultural script for both the causes and symptoms of hwa-byung in Korea such that it has the reputation of being “Korea’s national illness” (Suh, 2013, p. 82). The symptoms of hwa-byung are more or less scripted, allowing for the afflicted ones to communicate their distress with physical states and feel understood by others.

In sum, both neurasthenia of China and hwa-byung of Korea are somatic idioms of distress. In both cases, the cultural scripts are available to members of the two East Asian cultural contexts. These members have a collective knowledge about what it means to have or express physical symptoms when one is suffering. The present work focuses on the view that emotional expression is fundamentally interpersonal and unfolds in the sociocultural context. Somatization, too, at its core, is emotional communication and thus should be examined in the context of cultural meaning systems regarding emotions (Averill, 2012). The present study aims to show how somatization influences interpersonal consequences of distress communications among people in Korea and US. To this end, we first review the shared characteristics of East Asian cultures that are different from Western cultures.

### Cultural Differences in Emotional Expression and Suppression

East Asians’ tendency to emphasize somatic symptoms rather than emotional states in their communication can be understood in relation to their cultural norms for expressing emotions (Cheung, 1986; Kleinman, 1986; Ryder et al., 2012). Notably, paying attention to and maintaining social harmony is particularly crucial for East Asians who tend to endorse the interdependent model of self. Preventing potential risks to relational harmony becomes an important goal for individuals in interdependent cultural contexts. Thus, emotional restraint is a norm in this culture (Wierzbicka, 1995; Matsumoto and Fontaine, 2008). This may be especially true for cases of severe emotional disruptions like depression that trigger strong social stigma. Stigma in Asian culture has a particularly great impact in that mental illness not only reflects badly on the individual who is ill, but also on his or her family members (Littlewood et al., 2007). Consequently, East Asians tend to limit their emotional communication to their primary social networks including family members or close friends (Cheung et al., 1984). In comparison, in European American contexts where the independent model of self is endorsed, one’s behavior is largely contingent upon one’s own thoughts, feelings, and behaviors (Markus and Kitayama, 1991). In this context, open and explicit emotional expressions allow individuals to assert their unique sense of self. Thus, emotional expression is not only a societal norm but also a predictor of positive outcomes, such as subjective well-being in European American cultural context (Wierzbicka, 1992; Berenbaum and James, 1994; Sheldon et al., 1997).

Such differences in cultural norms for emotional expressions imply that there should be cultural differences in suppression of emotions. Emotional suppression in the West has been known to disrupt optimal intrapersonal and interpersonal functioning, causing distress and tension for both suppressors and their interaction partners (Keltner and Kring, 1998; Butler et al., 2007; Butler, 2011). However, emerging research suggests that emotional suppression may be normative or even functional in the East Asian context (Su et al., 2012; Le Bonnie and Impett, 2013). For individuals, who strongly endorse Asian values, suppression is associated with prosocial goals, such as preserving relationships, rather than with self-protective (and potentially interpersonally harmful) goal, such as being assertive (Butler et al., 2007). In sum, whereas emotional suppression goes against the norm of openly expressing emotion in the European American cultural context, it may acceptable in the East Asian cultural context to avoid expressing emotions to others in hopes of maintaining social harmony.

### Relational Concern in Help-Seeking

Cultural differences in social support-seeking reflect aforementioned cultural differences in emotional expression. Consistent with the tendency to avoid active disclosure of negative emotions in interpersonal contexts, East Asians are more reluctant to directly seek support from others than are European Americans (Taylor et al., 2004, 2007; Kim et al., 2006). For example, participants in one study reported their preferred strategies for dealing with stress (i.e., seeking emotional support, positive reframing, and denial). Participants with an Asian cultural background reported using social support, and particularly emotional support, less than did European Americans, when coping with stressful situations (Taylor et al., 2004). Examinations of possible explanations for this cultural difference revealed that Asian Americans, compared to European Americans, tended to show greater levels of relational concerns, that is, fear of negative consequences of seeking help in relationships (Taylor et al., 2004; Kim et al., 2006). Asian Americans tended to perceive help from others as less than effective. These finding are congruent with the East Asian model of interdependent self, because they reflect a greater emphasis on relational concerns.

### Somatization as an Affective Communication Strategy

Given East Asian cultural norms of emotional expression and seeking social support, the emphasis on somatic symptoms may be an effective strategy for securing social support and health resources in these cultural contexts (Cheung, 1986; Kleinman, 1986; Ryder et al., 2012). It is possible that the somatic scripts of communicating distress have been formed through socialization to reflect culture-specific incentives (D’Andrade, 1984; Kirmayer and Sartorius, 2007). Interpersonal reinforcement through verbal and non-verbal cues can shape how one talks about distressing experiences. Indeed, in a study conducted on American undergraduates, researchers have found that participants who received verbal social incentives (i.e., positive verbal reaction) after using either somatic or psychological words to describe distressing situations subsequently increased their use of the reinforced types of words (Lam et al., 2005). It does appear that East Asian contexts provide such incentives for somatic expressions of distress. According to Wu and Tseng (1985), Chinese mothers demonstrate care and love for their children by paying attention to their bodily need. Whereas expressing negative emotions like fear would not get a child much attention or may even result in getting scolded, complaints about physical aches and pains, such as abdominal pain, would prompt the mother to focus on the child, for example by giving the child some warm soup to eat. These examples suggest that somatization in East Asian cultural contexts may be effective in recruiting support and care from others.

## Summary and Research Overview

The present research adopts the perspective that somatic expression may serve as a form of functional communicative strategy in interpersonal contexts. First of all, such an effective communicative strategy might have a positive impact on the experience of a person who is expressing distress. It may relieve communicators’ apprehension about disrupting relational harmony, and make communication more free-flowing. This may contribute to communicators’ perception that they are communicating in an effective manner and that their interaction partner would empathize with them, resulting in heightened satisfaction with communication. Moreover, the effective communicative strategy may also bring about desirable reactions (e.g., support and empathy) from the interaction partner.

In sum, the primary aim of the present research is to examine cultural differences in the use of somatic expressions, particularly in their functions for people who are involved in the communication. Relational concerns about sharing negative experiences were taken into account. To our knowledge, the present work will be the first to test whether there are differential consequences of somatic expressions for communicators (people who discloses distress; Study 1) and for interaction partners (people who are the targets of distress disclosure) depending on the cultural context (Study 2). This study examines distress expressions in Korea and in the US, two cultural contexts that have been shown to differ in their emphasis on somatic symptoms.

## Study 1

### Communicative Effects of Somatization on a Communicator

In Study 1, participants wrote about their experiences of negative emotions addressing an imagined interaction partner. Considering that individuals’ behaviors depend on social partners (Goffman, 1967; Pennebaker et al., 2003), and that this is particularly important for those from interdependent cultural contexts (Kitayama and Markus, 2000), two different types of imagined interaction partner—a friend and a therapist—were examined. Specifically, participants were asked to write distress narratives either in the imagined presence of a close friend or a psychology professional (i.e., therapist, counselor). This method allowed us to examine individuals’ emotional descriptions as directed at a specific partner. Although hypothetical in nature, previous studies have found this method to be effective in deriving different language use depending on the interaction partner (Morand, 2000). Friends are thought of as one of the most common sources of companionship and support (Crohan and Antonucci, 1989), particularly for East Asians (Cheung et al., 1984). The other interaction partner, a therapist, was chosen given that previous findings on somatization were largely based on a client-expert (i.e., psychiatrist, counselor, or therapist) relationship.

There are various approaches to analyzing verbal behavior, focusing on different aspects of language. Due to its efficiency, a quantitative approach to text analysis has gained popularity among researchers interested in identifying psychological properties in language. The present study used the Linguistic Inquiry and Word Count (LIWC) for quantitative text analysis of the US participants’ narratives (Pennebaker et al., 2001). We predicted that Korean participants would use more somatic expressions than US participants. Moreover, we examined whether there are any cultural differences in the effects of somatic expressions on communicators’ perceived quality of disclosure. Given the cultural script of somatic expressions of distress in Korean culture, we predicted that somatization would have a positive effect for Koreans, such that the greater use of somatic expressions would be associated with increased perceived disclosure quality by a communicator. In contrast, in the absence of such somatic scripts in American culture, it was predicted that somatic expressions would not be associated with perceived disclosure quality for US communicators. Given the presence of “Western psychologization” (White, 1982; Kleinman, 1986; Ryder et al., 2008; but see Tsai et al., 2004 and Zhou et al., 2011), we also examined whether US participants would use more negative affect words than Koreans and further explored whether or not use of negative emotions would have different effects on perceived disclosure depending on cultures.

It was hypothesized that the perceived quality of disclosure would depend on the interaction partner for Koreans, who are likely to be more sensitive to interpersonal context (Matsumoto, 1993). Additionally, the preference for family members and friends over psychological professionals as confidants among Asians (Cheung, 1984) suggests that Koreans would view their disclosure to a friend as more satisfying than to a therapist. In contrast, given that European Americans’ emotional expressions are relatively less influenced by the interpersonal contexts (Matsumoto, 1993), it was predicted that US participants’ perceived disclosure quality would not differ depending on the interaction partner.

A final cultural factor relevant to perceived disclosure quality was relational concerns about help seeking. In help-seeking situations, one may be concerned about the negative consequences of help-seeking, such as burdening others, upsetting relationships, or losing face (Taylor et al., 2004). The present study explores the potentially moderating effect of culture on the relationship between relational concerns and perceived disclosure quality. Based on previous literature on cultural differences in relational concerns, we predicted that higher relational concerns would point to lower perceived disclosure quality for Koreans. In contrast, it is expected that the perceived disclosure quality of US participants would not be affected by the relational concerns.

### Method

#### Participants

There were 113 Korean participants (79 females and 34 males; 100% Koreans) and 119 US participants (58 females and 61 males; 82.4% White Americans, 10.1% African Americans, 5.0% Hispanic Americans, 3% other ethnicities). All Korean participants were born and raised in Korea. Korean participants were recruited from Kangwon National University, in Chuncheon City, South Korea. Korean participants participated in a group of 10 to 30 in a big classroom and completed paper-and-pencil questionnaire. US participants were recruited using Amazon.com’s Mechanical Turk (MTurk) interface and completed an online survey. They were restricted to those who reside in the US and are between the ages of 18 and 25. The age was restricted in order to make the US sample equivalent to Korean college student sample. In addition to its remarkable convenience in recruiting participants, MTurk has been documented to be a valid way of getting quality data (Berinsky et al., 2011). The independent samples *t*-test revealed that there was no difference in the SES of the family in which they grew up between Korean and US samples (1 = low income; 5 = upper income; *M*_Korea_ = 3.00, *SD*_Korea_ = 0.83; *M*_US_ = 2.82, *SD*_US_ = 0.87), *t*(223) = -1.55, *p* = 0.122. However, there was a significant difference in age between these two cultural groups, *t*(184.43) = -2.62, *p* = 0.009. Korean participants (*M* = 21.31, *SD* = 5.41) were significantly younger than US participants (*M* = 22.87, *SD* = 3.33). This is due to the fact that US sample from MTurk included both students (*n* = 58) and non-students (*n* = 61). There was no cultural difference in the SES between Korean and US samples regardless of whether US participants were student [*t*(162) = -1.20, *p* = 0.230] or non-student [*t*(165) = -1.38, *p* = 0.170]. Additional analyses were conducted to ensure that the main results for student and non-student group in US sample were equivalent.

### Procedure

First, participants completed the initial questionnaire which requested demographic information and initial levels of positive and negative affect. They were then provided with an instruction telling them to write about a situation in which they had conflict with other(s), were treated unfairly, and felt very badly. The purpose was to capture negative emotional states, specifically anger, which is the prototypical emotion associated with the Korean somatization syndrome, hwa-byung. The instruction purposefully avoided using the emotional words such as angry or upset in order to avoid potentially priming different meanings of the word in two languages. The participants were asked to write about their experience as if they were talking to either a friend or a therapist. The instruction for the writing task was as follows:

Think about a situation in the past 12 months in which you had a conflict with someone, were treated unfairly and felt very badly. Now imagine that you are talking about the experience in this situation to one of your friends/to a therapist. Think about how this situation affected you physically or psychologically (for example, how it made you think or feel). In the space below, I would like you to write what you would tell your friend/a therapist about this experience, as if you are actually talking to him/her in person. Ideally, the situation you are writing about should be the one that you have not previously discussed with others, or have been discussed to a minimal extent. Don’t worry about spelling, sentence structure or grammar. Also, your information will remain confidential, so feel free to express yourself openly.

The participants’ written narratives were then analyzed using the LIWC program which has been widely used in previous studies to detect a wide range of meaningful psychological processes including attentional focus, emotionality, and thinking styles (for a review, see Tausczik and Pennebaker, 2010). The LIWC operates based on a word count strategy. The narratives of Korean participants were analyzed using the Korean Linguistic Inquiry and Word Count (KLIWC, Lee et al., 2005). This study focused on two word categories: somatic and negative emotion words. Somatic words included physical symptoms or states (i.e., ache, heart), eating, and sleeping. Negative emotion words included anger, anxiety, and sadness (i.e., hurt, angry).

After writing about their negative experiences, participants completed a questionnaire asking about their perceived disclosure quality and relational concerns, in this order. The measurements assessing relational concerns were provided after the writing task to avoid the possible influence that these measurements may have on participants’ disclosure. All materials including the writing instruction and measurements followed the standard translation and back-translation procedures. The first author translated the English material into Korean. Then, a person who is bilingual and proficient in English and Korean back-translated the Korean version into English. Any discrepancy between the original version and back-translated English version was addressed through discussion between the translators to assure linguistic equivalence of the material.

### Materials

#### Initial Mood

The mood state of participants was assessed using the 10-item International Positive and Negative Affect Schedule Short Form (I-PANAS-SF; Thompson, 2007). Positive affect scale included items assessing the following states: alert, inspired, determined, attentive, and active. Negative Affect scale included items assessing the following states: upset, hostile, ashamed, nervous, and afraid. The items were measured on a 5-point scale ranging from 1 (Never) to 5 (Always). Cronbach’s alphas for positive affect were 0.60 and 0.83 for Korean and US participants, respectively. Cronbach’s alphas for negative affect were 0.79 and 0.83 for Korean and US participants.

#### Negative Impact of the Experience

Participants were asked to rate the degree of the negative impact of the experience described in the narratives. This measure included two items asking: “how serious was this experience?” and “how much distress did this experience cause you?” The items were measured on a 4-point scale ranging from 1 (*Not at all*) to 4 (*Very much*). The average score of these two items was used as a composite score of negative impact of the experience. Cronbach’s alphas for these two items were 0.71 for Korean and 0.67 for US participants.

#### Relational Concerns

Participants completed an 11-item questionnaire that captures the concerns that might affect whether or not they might seek social support from others (Kim et al., 2006). Participants were asked to rate how important each of the concerns would be for them in seeking social support on a scale of 1(*Not at all*) to 5 (*Very much*). The relational concerns items captured reasons for not seeking social support, such as desire to maintain the group harmony, belief that seeking help would make the problem worse, and concern for others’ evaluation and criticism when sharing problems. Cronbach’s alphas of this scale were 0.86 for Korean and 0.91 for US participants.

#### Perceived Disclosure Quality

After writing about their experience, participants were asked to report how they evaluated their experience of disclosing distress to the interaction partner. Participants’ perceived disclosure quality encompasses the perception of self-efficacy in communicating distress, the expectation of empathic response from the interaction partner, and general satisfaction with disclosure. This measure consisted of three items asking to report the interaction partner’s (a friend or a therapist) empathy for them, the extent to which their communication was effective, and the extent to which they were satisfied with their disclosure. These items were rated on a 4-point scale ranging from 1 (*Not at all*) to 4 (*Very much*) and were averaged to generate a composite score of perceived disclosure quality. Cronbach’s alphas for these three items were α = 0.86 for Korean and α = 0.81 for US participants.

#### LIWC Categories

Of the categories available in LIWC, eight word categories were relevant to the present study: negative emotion words (i.e., hate, nervous), anxiety words (i.e., tense, afraid), anger words (i.e., hate, pissed), sadness words (i.e., grief, cry, sad), physical state words (i.e., ache, breast), body state words (ache, heart, cough), eating words (eat, swallow, taste), and sleeping words (asleep, bed, dreams). Each LIWC variable represents the percentage of the number of words that belongs to the given category in a given text. The LIWC categories are arranged hierarchically; anxiety words, anger words, and sadness words are included in the superordinate category of negative emotion words. Likewise, body state words, eating words, and sleeping words categories are included in the superordinate category of physical state words. Studies using LIWC have reported good internal consistency across time, topic, and text source (Pennebaker and King, 1999; Mehl and Pennebaker, 2003). In addition, LIWC categories and human ratings of writing samples show high correlation, indicating high external validity of LIWC (Alpers et al., 2005).

Below is a sample of a disclosure narrative written by a Korean participant, as analyzed by LIWC.

“I have a master’s degree in international relations and I wanted to find a job that is relevant to what I studied. I took this job at a research institute where I get to work on relevant issues, however, it is a temporary position for 1 year and the pay is unbelievably low. Also, I am realizing now that the work is mostly administrative, boring, and so simple that even a high school graduate could manage it. Moreover, one coworker and I have been on bad terms since the beginning …. For some reason, this girl is jealous of me and talks about me behind my back, which is so upsetting. Low salary, dull tasks, and a mean coworker are enough reasons to quit the job but I have no other option at the moment but to stay here. Ever since I started this job I have had **indigestion** and every once in a while I **vomit**. Also, 1 day, my sister found a patchy **baldness** in my **hair**.” (bold letters indicate somatic words and underlined words indicate negative affect words according to LIWC).

### Results

#### Topics of the Disclosure Narratives

Disclosed experiences included: conflicts with friend(s) (betrayal, falling out; 42.3%_Korea_; 29.1%_US_), problems with family members (parents, siblings, spouses, and relatives; 16.5%_Korea_; 19.7%_US_), romantic conflict (break-up, infidelity, and argument; 8.2%_Korea_; 12.0%_US_), conflict with people at work (boss, colleagues; 12.4%_Korea_; 21.4%_US_), conflict with strangers (e.g., customers, shop owners, taxi drivers; 10.3%_Korea_; 7.7%_US_), and other (stress from low income, searching for jobs, etc.; 10.3%_Korea_; 10.3%_US_). Two additional categories that emerged only from the narratives created by Korean participants but not American participants were conflict with seniors (school seniors, 0.09%) and self-blame (blaming oneself in the situation, 0.05%).

#### Initial Mood

There was a significant cultural difference in the baseline mood. Koreans were lower in positive affect (*M*_Korea_ = 3.67, *SD*_Korea_ = 0.90; *M*_US_ = 4.37, *SD*_US_ = 1.26), *t*(213.21) = 4.89 and higher in negative affect, *p* < 0.001 (*M*_Korea_ = 2.34, *SD*_Korea_ = 1.08; *M*_US_ = 1.69, *SD*_US_ = 0.95), *t*(225) = -4.84, *p* < 0.001. The initial levels of positive affect and negative affect were controlled for in the analyses examining participants’ perceived disclosure quality.

#### Negative Impact of the Experiences

There was a cultural difference in the levels of negative impact of the experiences disclosed by the participants. Americans reported greater negative impact of their experience than did Koreans (*M*_Korea_ = 2.63, *SD*_Korea_ = 0.76; *M*_US_ = 3.03, *SD*_US_ = 0.71), *F*(3,228) = 19.51, *p* < 0.001, $\eta_{p}^{2}$ = 0.08. In addition, there was an effect of interaction partner condition. The negative impact of the experience was greater for participants in the therapist condition (*M* = 2.96, *SD* = 0.75) compared to those in the friend condition (*M* = 2.70, *SD* = 0.76), *F*(3,228) = 9.25, *p* = 0.003, $\eta_{p}^{2}$ = 0.040. There was no culture by interaction partner condition interaction, *F*(3,228) = 0.23, *p* = 0.634, $\eta_{p}^{2}$ = 0.001. Since the negative impact of emotional experience has been associated with greater needs to share with others in previous studies (Rimé et al., 1998), the level of negative impact of the experience was controlled for in the subsequent analyses along with the initial levels of positive affect and negative affect.

#### Relational Concerns in Help-Seeking

There was no significant difference in levels of relational concerns between Korean and US participants (*M*_Korea_ = 2.70, *SD*_Korea_ = 0.71; *M*_US_ = 2.87, *SD*_US_ = 0.91, *F*(1,222) = 2.74, *p* = 0.10, $\eta_{p}^{2}$ = 0.012). There was also no effect of the interaction partner condition (*M*_Friend_ = 2.74, *SD*_Friend_ = 0.75; *M*_Therapist_ = 2.83, *SD*_Therapist_ = 0.88, *F*(1,222) = 0.91, *p* = 0.342, $\eta_{p}^{2}$ = 0.004), nor the culture by interaction partner condition interaction, *F*(1,222) = 0.03, *p* = 0.857, $\eta_{p}^{2}$< 0.001.

### Somatic and Negative Affect Word Categories

In order to ensure that LIWC and KLIWC operate relatively equivalently, the structure of LIWC and KLIWC variables were examined by looking at the correlations between overarching categories (i.e., negative affect words and somatic words) and subcategories (i.e., negative emotion, anger, body, and eat) of interest. As is shown in the correlation tables, the relationship among the negative affect/somatic words subcategories between LIWC and KLIWC are highly similar (**Table 1**).

**Table 1:** Intercorrelations among the negative affect words and somatic words.

	1	2	3	4	5	6	7	8
(1) Negative emotion		0.28ˆ**	0.55ˆ***	0.29ˆ**	0.16	0.09	0.15	0.15
(2) Anxiety	0.54ˆ***	–	-0.17	0.10	0.00	0.05	-0.11	0.07
(3) Anger	0.67ˆ***	0.07	–	-0.08	0.19ˆ*	0.03	0.28ˆ**	0.21ˆ*
(4) Sad	0.31ˆ**	0.20ˆ*	-0.18ˆ*	–	-0.10	-0.12	0.04	-0.07
(5) Physical	0.14	0.18ˆ*	0.04	0.13	–	0.78ˆ***	0.40ˆ***	0.51ˆ***
(6) Body	0.12	0.12	0.07	0.05	0.73ˆ***	–	-0.01	0.23ˆ*
(7) Eat	-0.01	0.01	-0.03	0.01	0.69ˆ***	0.24ˆ*	–	0.14
(8) Sleep	0.24ˆ*	0.35ˆ***	-0.02	0.27ˆ**	0.22ˆ*	0.16	-0.06	–


*Intercorrelations for Korean participants are presented above the diagonal, and intercorrelations for US participants are presented below the diagonal. ^∗∗∗^*p* < 0.001, ^∗∗^*p* < 0.01, ^∗^*p* < 0.05, *p* < 0.10.*

#### Somatic Words

A series of 2 (culture: Korea, US) × 2 (interaction partner condition: therapist vs. friend) ANOVAs were conducted on the somatic word categories (physical state, body state, eating, and sleeping words). The main effects of interaction partner condition were not significant for any of the somatic words categories (all *F*s < 2.23). However, there was a significant main effect of cultural group for all somatic word categories. Consistent with the prediction, Korean participants were more likely than US participants to use physical state words, *F*(1,228) = 9.24, *p* = 0.003, $\eta_{p}^{2}$ = 0.039, body state words, *F*(1,228) = 22.30, *p* < 0.001, $\eta_{p}^{2}$ = 0.089, eating words, *F*(1,228) = 19.604, *p* = 0.000, $\eta_{p}^{2}$ = 0.079, and sleeping words, *F*(1,228) = 15.43, *p* < 0.001, $\eta_{p}^{2}$ = 0.063. There was no culture by partner condition interaction on any of the somatic word categories, (all *F*s < 0.64). **Table 2** presents the mean rates and standard deviations for each word category across the two cultural groups. Two interaction partner conditions were collapsed since there was no main effect or the interaction effect between interaction partner condition and culture for any of the word categories.

**Table 2:** Means and standard deviations of the percentage of the number of somatic and emotion words according to LIWC 2001.

	Korea (*N* = 113)		US (*N* = 119)		
			
	*M*	*SD*	*M*	*SD*	*F*(3,228)
Physical state	1.29	1.03	0.87	1.05	9.24ˆ**
Body state	0.84	0.77	0.42	0.65	22.30ˆ***
Sleep	0.21	0.34	0.07	0.20	15.43ˆ***
Eat	0.67	0.85	0.26	0.58	19.60ˆ***
Negative emotion	3.61	1.66	2.86	1.72	10.49ˆ**
Anxiety	0.45	0.60	0.46	0.75	0.02
Anger	1.19	1.16	1.14	1.08	0.20
Sad	0.53	0.59	0.51	0.59	0.05


*^∗∗∗^*p* < 0.001, ^∗∗^*p* < 0.01.*

#### Negative Affect Words

A series of 2 (culture: Korea, US) × 2 (interaction partner condition: therapist vs. friend) ANOVAs were conducted on negative affect word categories (negative emotion, anxiety, anger, and sadness). The main effect of interaction partner condition was not significant (all *F*s < 1.26). There was a main effect of culture for negative emotion words, *F*(1,228) = 10.49, *p* = 0.001, $\eta_{p}^{2}$ = 0.044. Korean participants used greater negative emotion words than US participants did, *F*(1,228) = 10.49, *p* < 0.01. However, there was no cultural group difference in anxiety [*F*(1,228) = 0.02, *ns*], anger [*F*(1,228) = 0.20, *ns*], and sadness words [*F*(1,228) = 0.05, *ns*]. There was no culture by interaction partner condition effect for any of the negative emotion word categories (all *F*s < 1.18; **Table 2**).

#### Perceived Disclosure Quality

**FIGURE 1:**
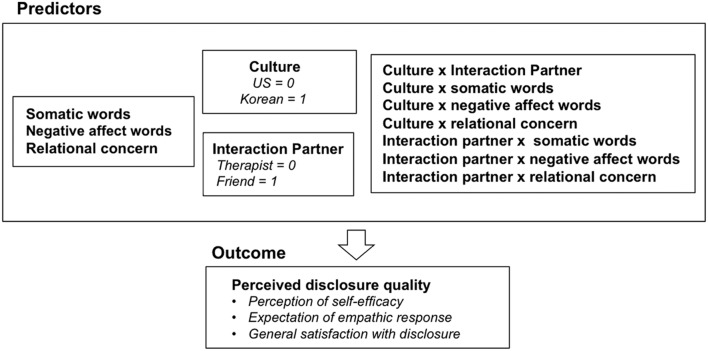
**The summary of the variables included in the hierarchical multiple regression model in Study 1**.

We conducted a hierarchical multiple regression to examine the role of interaction partner, somatic words, negative affect words, and relational concerns for disclosure quality for Korean and US participants. In doing so, we controlled for the initial mood and the impact of the experience. The scores of the initial mood, the impact of the experience, and relational concerns were used for these variables. Somatic words and negative affect words variables represent the percentage of the number of words that belong to the respective categories in a given text. Following Aiken and West (1991), all continuous variables were centered to reduce multicollinearity and interaction terms were calculated with these mean-centered variables. The variables that are included in the model are graphically presented in **Figure 1.**

We predicted a significant interaction between culture and other predictors on perceived disclosure quality. Specifically, Korean participants’ perceived disclosure quality was expected to vary depending on the interaction partner, somatic words, and relational concerns, whereas it was predicted that US participants would only be affected by negative affect words used in the disclosure. In Step 1, the baseline levels of positive and negative affect and the negative impact of the experiences were entered to control for these factors. In Step 2, dummy-coded interaction partner (therapist = 0; friend = 1), dummy-coded cultural group (US = 0; Korea = 1), somatic word use, negative affect word use, and relational concerns were entered. In Step 3, the interaction between the two dichotomous variables culture and interaction partner, and the interaction between these two dichotomous variables and all of the continuous variables, including somatic words, negative affect words, and relational concerns were entered. Higher interaction terms were not included in the model, since preliminary regression models have shown that they did not contribute to additional explained variance (Δ*R*^2^ = 0.004, Δ*F* = 0.673). Preliminary analyses showed no significant effects of gender or age, so these variables were dropped from the model.

The results from the hierarchical multiple regression analysis are summarized in **Table 3.** First of all, among the control variables, only baseline positive affect, *B* = 0.14, β = 0.21, *SE* = 0.04, *t* = 3.69, *p* < 0.001, but not the initial negative affect and the negative impact of the experience predicted perceived disclosure quality (Step 2). The main effect of culture was significant, indicating that US participants reported higher perceived disclosure quality than Koreans, *B* = -0.51, β = -0.35, *SE* = 0.10, *t* = -5.41, *p* < 0.001. In addition, interaction partner predicted perceived disclosure quality. Specifically, participants in friend condition reported higher perceived disclosure quality than those in therapist condition, *B* = 0.42, β = 0.28, *SE* = 0.08, *t* = 5.09, *p* < 0.001.

**Table 3:** Unstandardized regression coefficients predicting perceived disclosure quality.

	Step 1	Step 2	Step 3
Positive affect	0.19 (0.04)^∗∗∗^	0.14 (0.04)ˆ***	0.12 (0.04)ˆ***
Negative affect	-0.14 (0.04)^∗∗^	-0.05 (0.04)	-0.06 (0.04)
Impact of the experience	0.08 (0.06)	0.08 (0.06)	0.10 (0.06)
Interaction partner		0.42 (0.08)ˆ***	0.12 (0.11)
Culture		-0.51 (0.10)ˆ***	-0.85 (0.12)ˆ***
Somatic word		0.03 (0.04)	-0.01 (0.06)
Negative affect word		-0.02 (0.02)	0.01 (0.04)
Relational concerns		-0.11 (0.05)	0.02 (0.07)
Interaction partner × Somatic word			-0.13 (0.07)
Interaction partner × Negative affect word			-0.03 (0.05)
Interaction partner × Relational concerns			-0.05 (0.10)
Culture × Somatic word			0.21 (0.07)ˆ**
Culture × Negative affect word			0.01 (0.05)
Culture × Relational concerns			-0.34 (0.10)ˆ**
Culture × Interaction partner			0.67 (0.16)ˆ***

Δ*R*^2^		0.21^∗∗∗^	0.11^∗∗∗^

Total *R*^2^	0.14^∗∗∗^	0.34^∗∗∗^	0.44^∗∗∗^


*^∗∗∗^*p* < 0.001, ^∗∗^*p* < 0.01.*

More importantly, the results from Step 3 revealed that the effects of interaction partner condition, relational concerns, and somatic words on perceived disclosure quality were moderated by culture. There was a significant interaction between culture and interaction partner, *B* = 0.67, β = 0.36, *SE* = 0.16, *t* = 4.21, *p* < 0.001. There was a significant interaction between culture and somatic words, *B* = 0.21, β = 0.20, *SE* = 0.07, *t* = 2.80, *p* = 0.006. Finally, there was a significant interaction between culture and relational concerns, *B* = -0.34, β = -0.22, *SE* = 0.10, *t* = -3.44, *p* = 0.001. No other interaction terms were significant predictors for perceived disclosure quality. The effects of somatic words, negative affect words, and relational concerns were not moderated by interaction partner, *B*s < | 0.13| , *t*s < | 1.80| . In addition, there was no interaction between negative affect words and culture, *B* = 0.01, β = 0.01, *SE* = 0.05, *t* = 0.14, *p* = 0.889.

**FIGURE 2:**
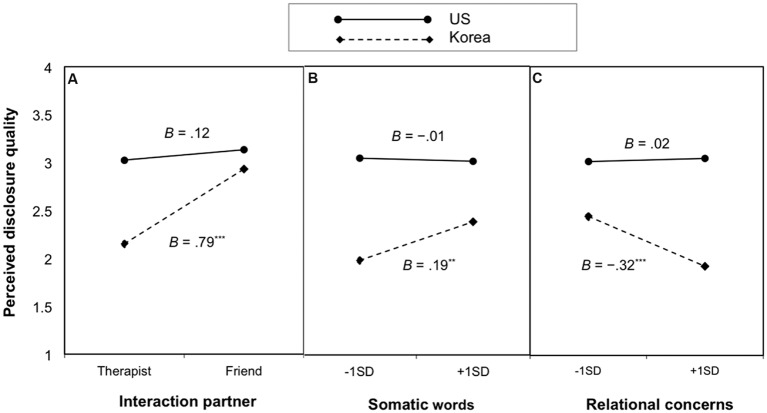
**Effects of **(A)** interaction partner, **(B)** somatic words, and **(C)** relational concerns on perceived disclosure quality for Korean and US participants in Study 1.** Estimated means for perceived disclosure quality are plotted at 1 SD lower and higher than the mean level of each of the three independent variables within each culture.

To probe the significant interaction effects, we used Hayes and Matthes’s (2009) MODPROBE SPSS macro and examined the effects of the predictors, interaction partner, somatic word use, and relational concerns on perceived disclosure quality depending on the moderator (culture). The analyses showed that the effect of interaction partner was significant for Korean participants, *B* = 0.79, *SE* = 0.11, *t* = 6.93, *p* < 0.001, but not for US participants, *B* = 0.12, *SE* = 0.11, *t* = 1.10, *p* = 0.272 (**Figure 2A**). Likewise, the effect of somatic words was only significant for Korean participants, *B* = 0.19, *SE* = 0.06, *t* = 3.04, *p* = 0.003, but not for US participants, *B* = -0.01, *SE* = 0.06, *t* = -0.24, *p* = 0.809 (**Figure 2B**). The effect of relational concerns was significant for Korean participants, *B* = -0.32, *SE* = 0.09, *t* = -3.59, *p* < 0.001, but not for US participants, *B* = 0.02, *SE* = 0.07, *t* = 0.26, *p* = 0.791 (**Figure 2C**).

Additionally, in order to examine whether student and non-student groups in US sample are comparable, the main analyses for both groups were run separately. Including only the student group of US sample resulted in the same pattern of results found for the sample as a whole. There was a significant effect of culture on perceived disclosure quality, with US participants reporting higher perceived disclosure quality than Korean participants, *B* = -0.87, β = -0.56, *SE* = 0.16, *t* = -5.70, *p* < 0.001. There was a significant culture by somatic words interaction, *B* = 0.24, β = 0.27, *SE* = 0.09, *t* = 2.55, *p* = 0.012, a significant culture by relational concerns interaction, *B* = -0.33, β = -0.22, *SE* = 0.14, *t* = -2.45, *p* = 0.015, and a significant culture by partner interaction, *B* = 0.63, β = 0.37, *SE* = 0.21, *t* = 2.99, *p* = 0.003. Likewise, including only the non-student group of US sample resulted in the same pattern. There was a significant effect of culture on perceived disclosure quality, with US participants reporting higher perceived disclosure quality than Korean participants, *B* = -0.78, β = -0.49, *SE* = 0.16, *t* = -4.79, *p* < 0.001. There was a marginally significant culture by somatic words interaction, *B* = 0.17, β = 0.19, *SE* = 0.10, *t* = 1.77, *p* = 0.079, a significant culture by relational concerns interaction, *B* = -0.31, β = -0.20, *SE* = 0.14, *t* = -2.22, *p* = 0.028, and a significant culture by interaction partner interaction, *B* = 0.65, β = 0.38, *SE* = 0.20, *t* = 3.22, *p* = 0.002.

### Discussion – Study 1

In Study 1, disclosure narratives of Korean and US participants were examined. Several findings are worthy of attention. First of all, the present study provided the first empirical evidence that Korean participants relied more on somatic expressions than did US participants when they were disclosing distressing experiences to friends and therapists. This adds to the previous empirical findings that documented a preponderance of somatic references in the communication of negative emotions in non-Western cultural contexts (e.g., Chinese, Ghanaian; Tsai et al., 2004; Dzokoto et al., 2013). Moreover, Koreans’ use of somatic words did not depend on the interaction partner, suggesting that the cultural script of somatization may not be limited to clinical settings, which were the focus of most prior studies. This is consistent with the recent findings suggesting that the cultural scripts of affective communication are characteristic of population as a whole and that clinical settings are only a part of a larger cultural context (Dzokoto et al., 2013).

Interestingly, Koreans used more negative emotion words in their disclosures than their US counterparts. As suggested by a recent review, somatization may not be so much a denial of emotional experience as emphasizing symptoms that are culturally salient and attention-worthy (Ryder et al., 2012). The findings from Study 1 support this view. It seems that the cultural differences in the communication strategy in distress disclosures did not lie in the emotional expressions but rather in the somatic expressions. Moreover, it may be worth noting that for Koreans somatic expressions may be working in concert with emotional expressions, given the co-occurrence of anger words, the most critical subcategory of negative affect words in this study, with physical state words (*r* = 0.19, *p* < 0.05), eating words(*r* = 0.28, *p* < 0.01), and sleeping words (*r* = 0.21, *p* < 0.05). In contrast, the anger words did not correlate with any of the somatic word categories for US participants (*r*s < 0.07).

Not only did Korean participants use more somatic words compared to US participants, these words predicted higher perceived disclosure quality for Korean participants. This result suggests that expressing negative emotions in a culturally endorsed way may have a “value from fit” (Higgins, 2000). Value from fit occurs when individuals pursue a goal activity with the means that match their habitual approach to goal pursuit. In the context of the present study, Koreans who communicated distress in a culturally shared way, by using somatic expressions, were also likely to perceive greater value of their communications. Whether or not Koreans employed physical terms in their communication with the intention of drawing empathy from others is difficult to know from this study. As for the negative affect words in the narratives, they had no significant contribution for disclosure quality in either cultural context. This is not surprising, considering prior findings that the use of negative emotion words did not have a simple linear relationship with psychological and health outcomes (Pennebaker et al., 1997; Pennebaker and Graybeal, 2001).

Moreover, perceived quality of disclosure depended on the interaction partner for Korean participants but not for US participants. As expected, Korean participants perceived their disclosure to be more effective, satisfying, and more empathy-worthy in their hypothetical communication with a friend compared to the one with a therapist. This result was consistent with previous research that has repeatedly found that Asians, compared to their US counterparts, are more reluctant to seek professional psychological help, underutilize formal mental health services, and end the treatment prematurely (Snowden and Cheung, 1990). This can also be explained by Asian cultural values emphasizing avoidance of shame and saving “face,” especially in interaction with people outside a close familial relationship (Zane and Yeh, 2002). Alternatively, it may be that Korean participants in the current study had limited previous experience with professional therapists, making it difficult for them to relive their moments of distress disclosure in such a context. On the contrary, US participants did not differ in perceived disclosure quality according to the interaction partner. This may be related to the Western cultural norms that encourage open emotional expression (Lutz, 1989) across different interpersonal contexts such as in public and in conversation with casual acquaintances (Matsumoto, 1993).

Finally, perceived disclosure quality was differentially influenced by relational concerns about help-seeking for Korean participants and for US participants. Although Korean and US participants were not different in their reports of the extent of their relational concerns about seeking help, Korean participants, but not for US participants, perceived their disclosure to be less effective and satisfying when they were concerned about the potentially negative consequences for the relationship in the process of seeking help. This adds to the previous research highlighting the role of relational concerns in explaining cultural difference in perceived benefits of social support (Taylor et al., 2004; Kim et al., 2008). Moreover, US participants’ positive evaluation of their own hypothetical disclosure irrespective of the interaction partner and relational concerns may reflect their cultural tendency for positive self-regard (Heine et al., 1999; Mezulis et al., 2004).

## Study 2

The results of Study 1 show that Korean and US participants disclose distress in different ways and believe that different factors make their communications more effective. But are they correct? Are each culture’s communication scripts more effective in producing desirable responses from listeners in the same culture? Answering these questions was the goal of Study 2.

When sharing negative emotions with others, people hold the expectation that others will react with support, thus promoting intimacy (Rimé, 2009). Indeed, emotional sharing brings about expected interpersonal consequences; individuals who detect signals of pain from others are motivated to engage in prosocial behaviors and feel compassionate for people in distress (see Batson et al., 2009, for a review). If somatization does serve as a functional script in a Korean cultural context, one may expect that somatic descriptions of experience would meet the communication goal, such as getting sympathy or help from others. Study 2 investigated whether Korean participants’ perceptions of positive interpersonal consequences of somatic narratives, based on Study 1’s observations, would correspond to actual positive reactions from potential interaction partners. Likewise, Study 2 examined whether the absence of positive relationship between somatic words and perceived disclosure quality for US participants found in Study 1 would correspond to the absence of positive reactions to somatic narratives from potential interaction partners. In sum, Study 2 examined the effects of different types of distress narratives for empathic responses of Korean and US participants. Specifically, participants in Study 2 were provided with distress narratives that included either emotional descriptions only or emotional descriptions with additional somatic descriptions signaling distress. Considering that, in Study 1, both Korean and US participants used negative affect words, we believe that including both emotional words and somatic words in the narrative would be more natural than providing purely somatic descriptions. We examined what empathic responses would be elicited by these types of narratives. We refer to these two conditions as affective and somato-affective conditions.

The present study focused on two distinct aspects of empathy-related responses, perspective taking and sympathy, as many researchers have identified these as the key aspects of empathic responses (Eisenberg, 1986, 2007). Perspective taking is defined as a cognitive ability to understand others’ mental state, including intentions, desires, and beliefs (Davis, 1983; Eisenberg et al., 1991), whereas sympathy, often used interchangeably with empathic concern, is considered to be an emotional response that involves feelings of sadness, concern for others, and compassion (Eisenberg, 2007). Although previous literature suggests that perspective taking is undoubtedly involved in the generation of altruistic behaviors such as sympathizing and helping (Eisenberg et al., 1994; Batson et al., 1997), it can have the opposite effect on empathy (Eisenberg, 2007). For example, taking the perspective of others may increase personal distress, which is a self-focused and aversive affective reaction, and thereby motivate a person to reduce one’s own distress by avoiding contact with the suffering others (Batson et al., 1991). Sympathy or empathic concerns, on the other hand, are closely linked to the action tendency to reduce the pain of others.

The narratives used in Study 2 described highly stressful experiences, such as difficulty with getting a job or conflicts with colleagues. Assuming that the results from Study 1 are grounded in social reality, it was expected that Korean participants would respond with more other-oriented empathic responses, especially sympathy, in response to the narratives with additional somatic descriptions compared to those with only emotional descriptions. As for US participants, since there was no expectation of positive relationship between somatic words and disclosure satisfaction based on Study 1, it was expected that there would be no difference in empathic responses between the two narrative conditions.

### Method

#### Participants

Eighty-four Korean participants (40 females, *M*_age_ = 22.49, *SD*_age_ = 2.45) were recruited from Korea University located in Seoul, Korea. Korean participants participated in groups of 10–30 in a big classroom and completed paper-and-pencil questionnaire. Seventy-nine European American (40 females, *M*_age_ = 23.35, *SD*_age_ = 2.45) participants were recruited in the S using Amazon’s Mechanical Turk system and completed an online survey. Korean participants were all born and raised in Korea. US participants were all born in the US and identified themselves as White Caucasian. There was a significant age difference between these two groups, *t*(160) = 2.42, *p* = 0.017. The US participants were older than Korean participants. However, we report the results of the present study without controlling for age, since our preliminary analyses showed that the statistical control of age did not alter our conclusions.

### Materials and Procedure

Participants were asked to read the narratives that described distressing experiences of anonymous writers. They then filled out a questionnaire asking about their responses to the narratives.

#### Narrative Stimuli

Participants were presented with two narratives that were created based on the typical descriptions provided in Study 1. The narratives were written in first-person, describing highly stressful recent experiences of a narrator. On narrative described looking for a job and the other described a conflict with a colleague. Each participant was randomized into either the affective narrative condition or the somato-affective narrative condition and was presented with two narratives of the same kind, with the narrative about searching for a job always presented first. All materials were written in English first and translated into Korean. The Korean version was back translated into English by a person who is fluent in English and Korean.

In the affective narrative condition, the experiences were described using only and affective words (e.g., “depressed,” “sad,” and “down”). In the somato-affective condition, physical terms were also used to describe the distressing experiences (e.g., “headache,” “dizziness,” and “stomachache”). Except for the last two sentences describing the narrators’ emotional and somatic states, the rest of the contents of the stories were identical. Below is an example of the narrative:

“When I was job searching, I felt I was doing all I could to find a job. I had a great resume, I thought I was interviewing well, and yet I still could not find a job for 8 months. In one particular interview, the interviewer was not only rude and condescending, and he also put me down about my education and experience. All I did was put my best foot forward, and I was treated very poorly. After this whole experience I felt very down and bad about myself. It was hard for me to go on my next interview. However, I did go on another interview but despite the fact that I fared much better, I was still very insecure about myself and my achievements. It was very hard to shake the feeling of insecurity and not feeling good enough. ***For months, I have been feeling very depressed and down almost all the time. I have been feeling really sad and depressed*** *(affective narrative).****For months, I have had constant acute headaches and dizziness. I haven’t been digesting food well so I lost a lot of weight*** *(somato-affective narrative).*

#### Manipulation Check

After reading the narratives, participants responded to a single item asking about how much physical distress they thought the writer went through. This measure was used as a manipulation check, on a scale from 1 (*not at all*) to 6 (*extremely*).

#### Negative Impact of the Experience

Participants were asked to indicate to what extent they thought the experiences in the narratives were serious and caused distress to the writers of each narrative on a scale from 1 (*not at all*) to 6 (*extremely*). Variable assessing the overall negative impact of the experience was created by averaging the responses of these items.

#### Sympathy

Participants responded to three items asking to indicate the extent to which they felt sorry for the writer, the extent to which they did not pity the writer (reverse coded), and the extent to which they wanted to help the writer on a scale from 1 (*not at all*) to 6 (*extremely*), (α_Korea_ = 0.84, α_US_ = 0.77).

#### Perspective Taking

The perspective taking measure was adapted from the perspective taking subscale of Davis’ (1983) Empathy Scales. The participants were asked to indicate to what degree they were able to: (1) relate to the situation the writers were in, (2) relate to the writers’ emotional state, (3) relate to the writers’ thoughts, on a scale from 1 (*not at all*) to 6 (*extremely*). The three items were averaged to create a composite score of perspective taking (α_Korea_ = 0.81, α_US_ = 0.87).

### Results and Discussion

#### Manipulation Check

A 2 (cultural group: Korea, US) × 2 (narrative type: affective, somato-affective) ANOVA was conducted on the item asking participants to indicate the perceived degree of the writer’s physical distress. There was a significant difference between the affective narrative (*M* = 2.66, *SD* = 1.33) and the somato-affective narrative (*M* = 4.52, *SD* = 1.00), *F*(3,159) = 114.83, *p* = 0.001, $\eta_{p}^{2}$ = 0.419. There was also a main effect of culture, *F*(3,159) = 12.47, *p* = 0.001, $\eta_{p}^{2}$ = 0.073. US participants (*M* = 3.91, *SD* = 1.36) perceived the writers to experience higher physical distress than Korean participants (*M* = 3.39, *SD* = 1.57). Finally, there was a significant interaction between narrative type and culture, *F*(1,159) = 5.68, *p* = 0.018. $\eta_{p}^{2}$ = 0.034, indicating that the difference in physical distress between affective and somato-affective narratives was more pronounced for Korean participants than for US participants. An examination of the effect of narrative type on this item for Korean and US participants separately revealed that across cultural groups participants perceived higher physical distress for somato-affective narratives, US participants, *t*(66.95) = -5.58, *p* < 0.001; Koreans, *t*(82) = -9.75, *p* < 0.001. Hence, our manipulation check indicated that the different usage of words successfully manipulated the writer’s physical distress perceived by the participants who read the affective narratives and those who read the somato-affective narrative.

#### Negative Impact of the Experience

A 2 (cultural group: Korea, US) × 2 (narrative type: affective, somato-affective) ANOVA was conducted to examine whether there was a cultural difference in the perceived negative impact of the experiences depending on the narrative type. The result of this ANOVA revealed a main effect of culture and a main effect of the narrative type, *F*(1,159) = 3.94, *p* = 0.049, $\eta_{p}^{2}$ = 0.024 and *F*(1,159) = 4.45, *p* = 0.037, $\eta_{p}^{2}$ = 0.027, respectively. US participants (*M* = 4.66, *SD* = 0.71) perceived the experiences described in the narratives to have greater negative impact on the writers than did Korean participants (*M* = 4.43, *SD* = 0.79) and participants who read somato-affective narratives (*M* = 4.65, *SD* = 0.76) perceived greater negative impact than those who read the affective narratives (*M* = 4.42, *SD* = 0.74). There was no culture by narrative type interaction effect, *F*(1,159) = 0.13, *ns.* Since reactions to emotional content are influenced by the intensity of emotions (Rimé, 2009), the subsequent analyses controlled for the perceived negative impact of the experience.

#### Sympathy

**FIGURE 3:**
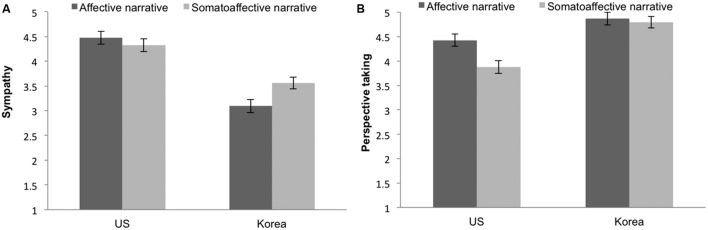
**(A)** Mean levels of sympathy and **(B)** perspective taking of Korean and US participants in emotion and somatic narrative conditions in Study 2.

To test whether there was a significant interaction between culture and narrative type on sympathy, we conducted a 2 (cultural group: Korea, US) × 2 (narrative type: affective, somato-affective) ANCOVA for experienced sympathy for the writers, controlling for the narratives’ negative impact of the experience. Overall, there was a significant effect of the covariate, negative impact of the experience of the narrative, on sympathy, *F*(1,158) = 49.37, *p* < 0.001, $\eta_{p}^{2}$ = 0.238. Furthermore, there was a significant main effect of culture, with US participants showing greater sympathy than Koreans, *F*(1,158) = 68.93, *p* < 0.001, $\eta_{p}^{2}$ = 0.304. However, no main effect of narrative type was observed, *F*(1,158) = 1.45, *p* = 0.231, $\eta_{p}^{2}$ = 0.009. Of importance, the interaction between culture and narrative type for sympathy proved significant, as predicted, *F*(1,157) = 5.70, *p* = 0.018, $\eta_{p}^{2}$ = 0.035 (see **Figure 3A**). As expected, follow-up comparisons revealed that Koreans responded with more sympathy for the somato-affective narratives (*M*_somato-affective_ = 3.56) compared to the emotional narratives (*M*_affective_ = 3.09), *F*(1,81) = 6.08, *p* = 0.016, $\eta_{p}^{2}$ = 0.070. On the other hand, US participants showed no difference in sympathy for the narrators in these two types of narratives (*M*_somatic_ = 4.33, *M*_affective_ = 4.47).

Additionally, in order to examine whether student and non-student groups in US sample are comparable, the main analyses for both groups were run separately. Including only the US student group sample resulted in the same pattern of results found for the sample as a whole. Although the interaction between culture and narrative condition did not reach statistical significance due to the reduced sample size (*F* = 3.20, *p* = 0.076), the same pattern of findings was observed. Similarly, the same pattern was observed for the analysis limited to the non-student group (*F* = 3.47, *p* = 0.065) although this pattern did not reach statistical significance due to the small sample size of non-student US participants.

#### Perspective Taking

The same 2 (cultural group: Korea, US) × 2 (narrative type: affective, somato-affective) ANCOVA was conducted for perspective taking, controlling for the negative impact of the experiences. Once again, there was a significant effect of the covariate, the negative impact of the experience, on perspective taking, *F*(1,158) = 39.61, *p* < 0.001, $\eta_{p}^{2}$ = 0.200. There also was a significant main effect of culture on perspective taking, *F*(1,158) = 28.97, *p* < 0.001, $\eta_{p}^{2}$ = 0.155, which was consistent with the prediction that East Asians were better at taking the perspective of others compared to US participants. There was a significant main effect of narrative condition on perspective taking, *F*(1,158) = 6.08, *p* = 0.015, $\eta_{p}^{2}$ = 0.037, with higher perspective taking for affective narratives than for the somato-affective narratives. There was a marginally significant interaction between culture and narrative condition, *F*(1,158) = 3.66, *p* = 0.058, $\eta_{p}^{2}$ = 0.023. Further analysis revealed that US participants reported greater perspective taking in response to affective narratives than to somato-affective narratives, *F*(1,76) = 7.90, *p* = 0.006, $\eta_{p}^{2}$ = 0.094, whereas there was no difference in perspective taking between the two narrative conditions for Korean participants, *F*(1,81) = 0.25, *p* = 0.618, $\eta_{p}^{2}$ = 0.003 (**Figure 3B**).

### Discussion – Study 2

Findings from Study 2 provided the first empirical evidence of cultural differences in the levels of pro-social emotions in response to distress narratives depending on the presence of somatic words in these narratives. The present findings partially supported the prediction that Koreans would respond to the somato-affective narratives with more other-oriented tendencies compared to the purely affective narratives. Koreans felt more sympathy in response to the distress narratives that included physical symptoms compared to the ones that focused exclusively on the explicit emotional terms. However, US participants felt similar levels of sympathy for these different styles of narratives. The belief in the effectiveness of somatic expressions as a communication strategy among Koreans (Study 1) appears to be grounded in the actual effectiveness of somatic expressions in inducing sympathetic reactions from others (Study 2).

One unexpected finding from Study 2 was the overall higher level of sympathy reported by US participants compared to Koreans. One possible explanation could be related to the differential sensitivity to others depending on the relationships one has with them in Korean culture. In a collectivistic cultural context, one’s prosocial emotional responses (e.g., sympathy) may be attenuated when one does not share any meaningful ingroup relationship with the target in distress (Kitayama and Markus, 2000). In contrast, the differences between ingroup and outgroup members are known to be attenuated in individualistic cultural contexts like the US. In this study, participants responded to the narratives of strangers, which may explain the lower levels of sympathy of Koreans relative to US participants. If the narratives were identified as coming from close others, the cultural difference in sympathy observed in the current study might have been reduced, if not reversed.

Interestingly, the patterns of cultural differences observed for perspective taking were different from those observed for sympathy—Koreans were higher in perspective taking compared to US participants. Moreover, the pattern for the effects of narrative condition on perspective taking was different from that of sympathy. Whereas Koreans showed uniformly high degrees of perspective taking across the two narrative conditions, US participants reported higher perspective taking in response to the affective narratives than to the somato-affective narratives. Koreans may be more attuned to the perspectives of others due to their cultural imperative to situate oneself in relation to others and focus attention on others’ actions and knowledge (Cohen et al., 2007; Wu and Keysar, 2007). However, for US participants, communications about feelings seem to be more effective for helping people relate to others’ difficult situations than communicating about feelings and the body.

## General Discussion

The present study extends prior literature by examining Korea, a context that shares cultural values with other East Asian cultures but is understudied on the topic of somatization. Korean hwa-byung provides a rich resource for future researchers interested in the cultural shaping of distress experience and expressions. At the outset of the present study, the act of sharing emotional experiences with others was hypothesized to be a communication strategy that may serve an adaptive function in a given culture. The findings of the present study suggest that the specific communicative strategies that are considered adaptive and are employed depend on cultural context. Like their Chinese or Chinese Canadians counterparts who showed a higher tendency to rely on somatic descriptions in communicating distress compared to North Americans (Kirmayer, 1989; Ryder et al., 2002; Ryder et al., 2012), Koreans relied on somatic descriptions to a greater degree than US participants in the present study. Evidence for the communicative value of somatization in Korean culture comes from the positive association between the somatic words used in the narratives and perceived disclosure quality in Study 1; Koreans who used more somatic words in communicating distress expected that others would react more positively to their stories. The accuracy of this expectation was confirmed by Korean observers’ greater levels of sympathy experiences in response to somato-affective descriptions in Study 2. In contrast, there was neither a positive association between somatic words and perceived disclosure quality nor greater sympathy in response to somato-affective rather than affective descriptions among US participants.

Unexpectedly, however, Koreans also used more negative affective words than US participants, which was inconsistent with the argument that Westerners are more likely than non-Westerners to have a “psychologization bias” (Parker et al., 2001). This pattern may be due to the fact that the Korean language is known to be rich in emotional words relative to other East Asian languages (Ahn et al., 1993). In addition, in a recent linguistic analysis of emotional metaphors in Korean and in English, Türker (2013) argued that the Korean metaphorical expressions of anger display features that are not observed in English. The present results suggest that Koreans may use as many emotional expressions as Americans when they are communicating anger-associated distress. Additionally, as mentioned briefly, somatization does not seem to preclude emotional expressions, but is rather characterized as the emphasis on the somatic symptoms (Ryder et al., 2012; Dere et al., 2013). In particular, in Korea, somatic expressions may be used in addition to emotional expressions to amplify to maximize the effects of disclosure.

### Limitations and Future Direction

Several shortcomings of the present work should be noted. First, in Study 1 participants’ verbal behaviors were studied using a hypothetical scenario and relying on written narratives rather than during the real-time interaction with real partners. The methodology of Study 1 undermines the generalizability of the present findings to actual emotional communication with others that may involve non-verbal behaviors such as facial expressions (Ekman and Rosenberg, 2005), body posture and movement (Keltner, 1995; Atkinson et al., 2004), and vocal tones (Banse and Scherer, 1996). A second limitation pertains to the inherent problem of the text analysis approach. The analysis of word use in written language relies on word counts and is a probabilistic system (Pennebaker and Graybeal, 2001). Thus, like any other text analysis program, LIWC analysis that was used in Study 1 does not account for the context, irony, sarcasm, or multiple meanings of the homonyms. Third, participants in Study 2 were given written narratives of unknown individuals describing their negative experiences. People’s empathic responses to strangers may be different from responses toward close others. It would be important to examine potential cultural differences in the relationship between communication strategy and empathy-related responses while taking relational closeness into consideration.

Another noteworthy issue that arises when researchers compare responses in different languages pertains to the inherent difficulty of determining whether we are examining language, culture, or both. While one prior study suggests that somatization may be primarily due to culture (Tsai et al., 2004), other studies indicate that language can shape somatic expressions used in emotional discussions (Dzokoto and Okazaki, 2006; Dzokoto et al., 2013). Language and culture are often considered so intricately interwoven that they may not be meaningfully separated from one another. Further research examining bilinguals in different cultural contexts (e.g., Anglophones in the US versus West Africa, India or Hong Kong), would provide additional information as to the relative impact of language and culture on distress communication.

## Conclusion and Implications

These limitations notwithstanding, the current work examined the long-standing puzzle of cross-cultural differences in somatization. Although previous researchers have posited that somatization may be strategic in nature (Kleinman, 1982, 1986; Kirmayer and Young, 1998), no empirical research has been conducted to examine the adaptive functions of somatic communications in different cultural contexts. The present study contributes to the existing literature on the cultural differences in emotional expressing, with a particular focus on the communicative functions of somatic expressions of distress in interpersonal relationships. Somatization has long been considered to be dysfunctional in Western cultural contexts, with their emphasis on the direct expressions of feelings (Keyes and Ryff, 2003). Findings from the present work suggest that, depending on the culturally shared beliefs and social reality formed through these beliefs, somatization may actually be beneficial in interpersonal interactions. The results of the present work unearth differences in emotional communication in Korean and American cultural contexts. It would be important for future researchers to consider that emotional expression and its relational consequences are moderated by cultural context.

In addition to aiding scientific understanding, these findings have the potential to be useful in clinical or consulting settings, particularly those that involve cross-cultural interactions. In these settings, the dominant mode of distress communication is characterized by Western psychologization. While Western-trained clinicians may readily identify mood symptoms in patients’ reports and provide appropriate diagnoses based on these symptoms, they may fail to notice that other symptoms, such as somatic ones, require attention. Moreover, during psychotherapy, clinicians trained in Western cultural context are likely to reinforce the tendency of their clients to report symptoms that fit well with their diagnostic system, which emphasizes emotional and psychological symptoms rather than somatic ones. Indeed, verbal operant conditioning is known to operate during psychotherapeutic interviews (Beach, 1989). The therapeutic approach of reinforcing discussion of psychological processes may work well for individuals who are familiar with this mode of discourse, fostering rapport between a therapist and a client and contributing to positive treatment outcomes. However, the same approach may not be effective for individuals from different cultural backgrounds in which discussions of emotions are not considered particularly useful or appropriate. Indeed, it is not uncommon for Koreans to consider talk therapy as not only foreign but pointless, and to prefer medications; only highly educated Koreans who are familiar with Western psychological concepts feel comfortable with psychological forms of therapy (McDonald, 2011).

Previous work suggests that emotional experiences and expressions are shaped by cultural contexts (Fehr and Russell, 1991; Kitayama et al., 2000; Mesquita and Karasawa, 2002). Through fostering countless repeated transactions between their members (Kim and Markus, 1999), cultural contexts shape individuals’ beliefs of how others might respond and the specific communicative strategies that are adaptive in a particular cultural environment. These cyclic processes linking social reality, cultural beliefs, and behavioral strategies constitute and sustain the cultural scripts of communicating negative emotional experiences. The utility that somatic expressions have for communicating distress in interpersonal contexts may be one of the factors that sustain cultural script like hwa-byung in Korea. The present study provides a possible mechanism underlying cultural shaping of distress communications.

## Author Contributions

EC was the principle investigator of this project and managed from the conception to completion of the study. YC-D was the graduate advisor of DC and consulted with the first author throughout the study. WGP gave insightful feedback regarding the interpretation of the data and construction of the manuscript.

## Conflict of Interest Statement

The authors declare that the research was conducted in the absence of any commercial or financial relationships that could be construed as a potential conflict of interest.

## References

[B1] Aiken L. S. West S. G. (1991). *Multiple Regression: Testing and Interpreting Interactions.* Newbury Park, CA: Sage.

[B2] Ahn S. H. Lee S. H. Kwon O. S. (1993). Activation dimension: a mirage in the affective space? *Korean J. Psychol. Soc. Pers.* 7 107–123.

[B3] Ainsworth M. D. S. Blehar M. C. Waters E. Wall S. (1978). *Patterns of Attachment: A Psychological Study of the Strange Situation.* Hillsdale, MI: Erlbaum.

[B4] Alpers G. W. Winzelberg A. J. Classen C. Roberts H. Dev P. Koopman C. (2005). Evaluation of computerized text analysis in an internet breast cancer support group. *Comput. Hum. Behav.* 21 361–376. 10.1016/j.chb.2004.02.008

[B5] Atkinson A. P. Dittrich W. H. Gemmell A. J. Young A. W. (2004). Emotion perception from dynamic and static body expressions in point-light and full-light displays. *Perception* 33 717–746. 10.1068/p5096 15330366

[B6] Averill J. R. (2012). The future of social constructionism: introduction to a special section of emotion review. *Emot. Rev.* 4 215–220. 10.1177/1754073912439811

[B7] Banse R. Scherer K. (1996). Acoustic profiles in vocal emotion expression. *J. Pers. Soc. Psychol.* 70 614–636. 10.1037/0022-3514.70.3.614 8851745

[B8] Batson C. D. Ahmad N. Lishner D. A. (2009). “Empathy and altruism,” in *Handbook of Positive Psychology*, eds Snyder C. R. Lopez S. J. (New York, NY: Oxford University Press), 485–498.

[B9] Batson C. D. Batson J. G. Slingsby J. K. Harrell K. L. Peekna H. M. Todd R. M. (1991). Empathic joy and the empathy-altruism hypothesis. *J. Pers. Soc. Psychol.* 61 413–426. 10.1037/0022-3514.61.3.413 1941512

[B10] Batson C. D. Early S. Salvarani G. (1997). Perspective taking: imagining how another feels versus imaging how you would feel. *Pers. Soc. Psychol. Bull.* 23 751–758. 10.1177/0146167297237008

[B11] Beach D. A. (1989). “The behavioral interview,” in *Clinical and Diagnostic Interviewing*, ed. Craig R. J. (Northvale, NJ: Jason Aronson), 79–93.

[B12] Berenbaum H. James T. (1994). Correlates and retrospectively reported antecedents of alexithymia. *Psychosom. Med.* 56 353–359. 10.1097/00006842-199407000-00011 7972618

[B13] Berinsky A. J. Huber G. A. Lenz G. S. (2011). Using mechanical turk as a subject recruitment tool for experimental research. *Polit. Anal.* 20 351–368. 10.1093/pan/mpr057

[B14] Bowlby J. (1969). *Attachment and Loss: Attachment*, Vol. 1 New York, NY: Basic Books

[B15] Bromet E. Andrade L. H. Sampson N. A. Alonso J. de Girolamo G. de Graaf R. (2011). Cross-national epidemiology of DSM-IV major depressive episode. *BMC Med.* 9:90 10.1186/1741-7015-9-90 PMC3163615 21791035

[B16] Butler E. A. (2011). Temporal interpersonal emotion systems: the ‘TIES’ that form relationships. *Pers. Soc. Psychol. Rev.* 15 367–393. 10.1177/1088868311411164 21693670

[B17] Butler E. A. Lee T. L. Gross J. J. (2007). Emotion regulation and culture: are the social consequences of emotion suppression culture-specific? *Emotion* 7 30–48. 10.1037/1528-3542.7.1.30 17352561

[B18] Cheung F. M. (1984). Preferences in help-seeking among chinese students. *Cult. Med. Psychiatry* 8 371–380. 10.1007/BF00114663 6499507

[B19] Cheung F. M. (1986). “Psychopathology among Chinese people,” in *The Psychology of the Chinese People*, ed. Bond M. H. (Hong Kong: Oxford University Press), 171–212.

[B20] Cheung F. M. Lau B. W. K. Wong S. W. (1984). Paths to psychiatric care in Hong Kong. *Cult. Med. Psychiatry* 8 207–228. 10.1007/BF00055168 6488844

[B21] Chiu C.-Y. Gelfand M. J. Yamagishi T. Shteynberg G. Wan C. (2010). Intersubjective culture: the role of intersubjective perceptions in cross-cultural research. *Perspect. Psychol. Sci.* 5 482–493. 10.1177/1745691610375562 26162194

[B22] Cohen D. Hoshino-Browne E. Leung A. K.-Y. (2007). “Culture and the structure of personal experience: insider and outsider phenomenologies of the self and social world,” in *Advances in Experimental Social Psychology*, ed. Zanna M. P. (Amsterdam: Elsevier), 1–67.

[B23] Crohan S. E. Antonucci T. C. (1989). “Friends as a source of social support in old age,” in *Older Adult Friendship: Structure and Process*, eds Adams R. G. Rosemary B. (Thousand Oaks, CA: Sage Publications), 129–146.

[B24] D’Andrade R. G. (1984). “Cultural meaning systems,” in *Culture Theory: Essays on Mind, Self, and Emotion*, eds Shweder R. A. LeVine R. A. (New York, NY: Cambridge University Press), 88–119.

[B25] Davis M. H. (1983). Measuring individual differences in empathy: evidence for a multidimensional approach. *J. Pers. Soc. Psychol.* 44 113–126. 10.1037/0022-3514.44.1.113

[B26] Dere J. Sun J. Zhao Y. Persson T. J. Zhu X. Yao S. (2013). Beyond ‘somatization’ and ‘psychologization’: symptom-level variation in depressed Han Chinese and Euro-Canadian outpatients. *Front. Psychol.* 4:377 10.3389/fpsyg.2013.00377 PMC3694214 23818884

[B27] Diagnostic and Statistical Manual of Mental Disorders [DSM-IV] (1994). *Diagnostic and Statistical Manual of Mental Disorders [DSM-IV]*, 4th Edn Washington, DC: American Psychiatric Association.

[B28] Dzokoto V. A. Okazaki S. (2006). Happiness in the eye and the heart: somatic referencing in West African emotion lexica. *J. Black Psychol.* 32 17–140. 10.1177/0095798406286799

[B29] Dzokoto V. A. Opare-Henaku A. Kpobi L. A. (2013). Somatic referencing and psychologisation in emotion narratives: a USA-Ghana comparison. *Psychol. Dev. Soc.* 25 311–331. 10.1177/0971333613500875

[B30] Eisenberg N. (1986). *Altruism, Emotion, Cognition, and Behavior.* Hillsdale, NJ: Lawrence Erlbaum.

[B31] Eisenberg N. (2007). *Empathy-Related Responding and Prosocial Behaviour.* London: Novartis Foundation Symposium. 17214311

[B32] Eisenberg N. Fabes R. A. Murphy B. Karbon M. Maszk P. Smith M. (1994). The relations of emotionality and regulation to dispositional and situational empathy-related responding. *J. Pers. Soc. Psychol.* 66 776–797. 10.1037/0022-3514.66.4.776 8189352

[B33] Eisenberg N. Fabes R. A. Schaller M. Miller P. Carlo G. Poulin R. (1991). Personality and socialization correlates of vicarious emotional responding. *J. Pers. Soc. Psychol.* 61 459–470. 10.1037/0022-3514.61.3.459 1941517

[B34] Ekman P. Rosenberg E. L. (2005). *What the Face Reveals: Basic and Applied Studies of Spontaneous Expression Using the Facial Action Coding System (FACS)*, 2nd Edn New York, NY: Oxford University Press.

[B35] Fehr B. Russell J. A. (1991). The concept of love viewed from a prototype perspective. *J. Pers. Soc. Psychol.* 60 425–438. 10.1037//0022-3514.60.3.425

[B36] Goffman E. (1967). *Interaction Ritual.* New York, NY: Doubleday Anchor.

[B37] Hayes A. F. Matthes J. (2009). Computational procedures for probing interactions in OLS and logistic regression: SPSS and SAS implementation. *Behav. Res. Methods* 41 924–936. 10.3758/BRM.41.3.924 19587209

[B38] Heine S. J. Takata T. Lehman D. R. (1999). Beyond self-presentation: evidence for self-criticism among Japanese. *Pers. Soc. Psychol. Bull.* 26 71–78. 10.1177/0146167200261007

[B39] Higgins E. T. (2000). Making a good decision: value from fit. *Psychol. Sci.* 30 1217–1230. 11280936

[B40] Keltner D. (1995). Signs of appeasement: evidence for the distinct displays of embarrassment, amusement, and shame. *J. Pers. Soc. Psychol.* 68 441–454. 10.1037/0022-3514.68.3.441

[B41] Keltner D. Kring A. M. (1998). Emotion, social function, and psychopathology. *Rev. Gen. Psychol.* 2 320–342. 10.1037//1089-2680.2.3.320

[B42] Keyes C. L. M. Ryff C. D. (2003). Somatization and mental health: a comparative study of the idiom of distress hypothesis. *Soc. Sci. Med.* 57 1833–1845. 10.1016/S0277-9536(03)00017-0 14499509

[B43] Kim H. Markus H. R. (1999). Deviance or uniqueness, harmony or conformity? a cultural analysis. *J. Pers. Soc. Psychol.* 77 785–800. 10.1037/0022-3514.77.4.785

[B44] Kim H. Sherman D. K. Ko D. Taylor S. E. (2006). Pursuit of comfort and pursuit of harmony: culture, relationships, and social support seeking. *Pers. Soc. Psychol. Bull.* 32 1595–1607. 10.1177/0146167206291991 17122173

[B45] Kim H. S. Sherman D. K. Taylor S. E. (2008). Culture and social support. *Am. Psychol.* 63 518–526. 10.1037/0003-066X 18793039

[B46] Kirmayer L. J. (1989). Cultural variations in the response to psychiatric disorders and emotional distress. *Soc. Sci. Med.* 29 327–339. 10.1016/0277-9536(89)90281-5 2669146

[B47] Kirmayer L. J. Sartorius N. (2007). Cultural models and somatic syndromes. *Psychosom. Med.* 69 832–840. 10.1097/PSY.0b013e31815b002c 18040090

[B48] Kirmayer L. J. Young A. (1998). Culture and somatization: clinical, epidemiological, and ethnographic perspectives. *Psychosom. Med.* 60 420–430. 10.1097/00006842-199807000-00006 9710287

[B49] Kitayama S. Markus H. R. (2000). “The pursuit of happiness and the realization of sympathy: cultural patterns of self, social, relations, and well-being,” in *Culture and Subjective Well-Being*, eds Diener E. D. Suh Eunkook M. (Cambridge, MA: The MIT Press), 113–161.

[B50] Kitayama S. Markus H. R. Kurokawa M. (2000). Culture, emotion, and well-being: good feelings in Japan and the United States. *Cogn. Emot.* 14 93–124. 10.1080/026999300379003

[B51] Kleinman A. (1982). Neurasthenia and depression: a study of somatization and culture in China. *Cult. Med. Psychiatry* 6 117–190. 7116909 10.1007/BF00051427

[B52] Kleinman A. (1986). *Social Origins of Distress and Disease.* New Haven, CT: Yale University Press.

[B53] Lam K. Marra C. Salzinger K. (2005). Social reinforcement of somatic versus psychological description of depressive events. *Behav. Res. Ther.* 43 1203–1218. 10.1016/j.brat.2004.09.003 16005706

[B54] Le Bonnie M. Impett E. A. (2013). When holding back helps: suppressing negative emotions during sacrifice feels authentic and is beneficial for highly interdependent people. *Psychol. Sci.* 24 1809–1815. 10.1177/0956797613475365 23846717

[B55] Lee C. H. Shim J. Yoon A. (2005). The review about the development of Korean linguistic inquiry and word count. *Korean J. Cogn. Sci.* 16 93–121.

[B56] Littlewood R. Jadhav S. Ryder A. G. (2007). A cross-national study of the stigmatization of severe psychiatric illness: historical review, methodological considerations and development of the questionnaire. *Transcult. Psychiatry* 44 171–202. 10.1177/1363461507077720 17576725

[B57] Lutz C. (1989). *Unnatural Emotions: Everyday Sentiments on a Micronesian Atoll and their Challenge to Western Theory.* Chicago, IL: University of Chicago Press.

[B58] Markus H. R. Kitayama S. (1991). Culture and the self: implications for cognition, emotion, and motivation. *Psychol. Rev.* 98 224–253. 10.1037/0033-295X.98.2.224

[B59] Matsumoto D. (1993). Ethnic differences in affect intensity, emotion judgments, display rule attitudes, and self-reported emotional expression in an American sample. *Motiv. Emot.* 17 107–123. 10.1007/BF00995188

[B60] Matsumoto D. Fontaine J. (2008). Mapping expressive differences around the world: the relationship between emotional display rules and individualism versus collectivism. *J. Cross Cult. Psychol.* 39 55–74. 10.1177/0022022107311854

[B61] McDonald M. (2011). Stressed and depressed, Koreans avoid therapy. *New York Times.* Available at: http://www.nytimes.com (accessed July 6, 2011).

[B62] Mehl M. R. Pennebaker J. W. (2003). The sounds of social life: a psychometric analysis of students’ daily social environments and natural conversations. *J. Pers. Soc. Psychol.* 84 857–870. 10.1037/0022-3514.84.4.857 12703653

[B63] Mesquita B. Albert D. (2007). “The cultural regulation of emotions,” in *The Handbook of Emotion Regulation*, ed. Gross J. J. (New York, NY: Guilford Press), 486–503.

[B64] Mesquita B. Karasawa M. (2002). Different emotional lives. *Cogn. Emot.* 16 127–141. 10.1080/0269993014000176

[B65] Mezulis A. H. Abramson L. Y. Hyde J. S. Hankin B. L. (2004). Is there a universal positivity bias in attributions? a meta-analytic review of individual, developmental, and cultural differences in the self-serving attributional bias. *Psychol. Bull.* 130 711–747. 10.1037/0033-2909.130.5.711 15367078

[B66] Min S. K. Suh S.-Y. Song K.-J. (2009). Symptoms to use for diagnostic criteria of hwa-byung, an anger syndrome. *Psychiatry Investig.* 6 7–12. 10.4306/pi.2009.6.1.7 PMC2796033 20046367

[B67] Morand D. A. (2000). Language and power: an empirical analysis of linguistic strategies used in superior-subordinate communication. *J. Organ. Behav.* 21 235–248. 10.1002/(SICI)1099-1379(200005)21:3<235::AID-JOB9>3.0.CO;2-N

[B68] Parker G. Gladstone G. Chee K. T. (2001). Depression in the planet’s largest ethnic group: the Chinese. *Am. J. Psychiatry* 158 857–864. 10.1176/appi.ajp.158.6.857 11384889

[B69] Pennebaker J. W. Francis M. E. Booth R. J. (2001). *Linguistic Inquiry Word Count.* Mahwah, NJ: Erlbaum.

[B70] Pennebaker J. W. Graybeal A. (2001). Patterns of natural language use: disclosure, personality, and social integration. *Curr. Dir. Psychol. Sci.* 10 90–93. 10.1111/1467-8721.00123

[B71] Pennebaker J. W. King L. A. (1999). Linguistic styles: language use as an individual difference. *J. Pers. Soc. Psychol.* 77 1296–1312. 10.1037/0022-3514.77.6.1296 10626371

[B72] Pennebaker J. W. Mayne T. J. Francis M. E. (1997). Linguistic predictors of adaptive bereavement. *J. Pers. Soc. Psychol.* 72 863–871. 10.1037/0022-3514.72.4.863 9108699

[B73] Pennebaker J. W. Mehl M. R. Niederhoffer K. G. (2003). Psychological aspects of natural language. Use: our words, our selves. *Annu. Rev. Psychol.* 54 547–577. 10.1146/annurev.psych.54.101601.145041 12185209

[B74] Preston S. D. de Waal F. B. M. (2002). Empathy: its ultimate and proximate bases. *Behav. Brain Sci.* 25 1–20; discussion 20–71. 12625087 10.1017/s0140525x02000018

[B75] Reis H. T. Patrick B. C. (1996). “Attachment and intimacy: component processes,” in *Social Psychology: Handbook of Basic Principles*, eds Higgins E. T. Kruglanski A. W. (New York, NY: Guilford Press), 523–563.

[B76] Rimé B. (2009). Emotion elicits the social sharing of emotion: theory and empirical review. *Emot. Rev.* 1 60–85. 10.1177/1754073908097189

[B77] Rimé B. Finkenauer C. Luminet O. Zech E. Philippot P. (1998). “Social sharing of emotion: new evidence and newquestions,” in *European Reviewof Social Psychology*, eds Stroebe W. Hewstone M. (Chichester: Wiley), 146–189.

[B78] Ryder A. G. Ban L. M. Chentsova-Dutton Y. E. (2011). Towards a cultural – clinical psychology. *Soc. Personal. Psychol. Compass* 5 960–975. 10.1111/j.1751-9004.2011.00404.x

[B79] Ryder A. G. Chentsova-Dutton Y. E. (2012). Depression in cultural context: “Chinese somatization”, revisited. *Psychiatr. Clin. North Am.* 35 15–36. 10.1016/j.psc.2011.11.006 22370488

[B80] Ryder A. G. Yang J. Heine S. J. (2002). Somatization vs. psychologization of emotional distress: a paradigmatic example for cultural psychopathology. *Psychology* 10 0–11.

[B81] Ryder A. G. Yang J. Zhu X. Yao S. Yi J. Heine S. J. (2008). The cultural shaping of depression: somatic symptoms in China, psychological symptoms in North America? *J. Abnorm. Psychol.* 117 300–313. 10.1037/0021-843X.117.2.300 18489206

[B82] Schachter S. (1959). *The Psychology of Affiliation.* Stanford, CA: Stanford University Press.

[B83] Shaver P. Klinnert M. (1982). “Schachter’s theories of affiliation and emotions: implications of developmental research,” in *Review of Personality and Social Psychology* Vol. 3 ed. Wheeler L. (Beverly Hills, CA: Sage), 37–71.

[B84] Sheldon K. M. Ryan R. M. Rawsthorne L. J. Ilardi B. (1997). Trait self and true self: cross-role variation in the Big-Five personality traits and its relations with psychological authenticity and subjective well-being. *J. Pers. Soc. Psychol.* 73 1380–1393. 10.1037/0022-3514.73.6.1380

[B85] Snowden L. R. Cheung F. K. (1990). Use of inpatient mental health services by members of ethnic minority groups. *Am. Psychol.* 45 347–355. 10.1037/0003-066X.45.3.347 2310083

[B86] Su J. C. Lee R. M. Oishi S. (2012). The role of culture and self-construal in the link between expressive suppression and depressive symptoms. *J. Cross Cult. Psychol.* 44 316–331. 10.1177/0022022112443413

[B87] Suh S. (2013). Stories to be told: Korean doctors between hwa-byung (fire-illness) and depression, 1970-2011. *Cult. Med. Psychiatry* 37 81–104. 10.1007/s11013-012-9291-x 23229388 PMC3585958

[B88] Tausczik Y. R. Pennebaker J. W. (2010). The psychological meaning of words: LIWC and computerized text analysis methods. *J. Lang. Soc. Psychol.* 29 24–54. 10.1177/0261927X09351676

[B89] Taylor S. E. Sherman D. K. Kim H. Jarcho J. Takagi K. Dunagan M. S. (2004). Culture and social support: who seeks it and why? *J. Pers. Soc. Psychol.* 87 354–362. 10.1037/0022-3514.87.3.354 15382985

[B90] Taylor S. E. Welch W. T. Kim H. Sherman D. K. (2007). Cultural differences in the impact of social support on psychological and biological stress responses. *Psychol. Sci.* 18 831–837. 10.1111/j.1467-9280.2007.01987.x 17760781

[B91] Thompson E. R. (2007). Development and validation of an internationally reliable short-form of the positive and negative affect schedule (PANAS). *J. Cross Cult. Psychol.* 38 227–242. 10.1177/0022022106297301

[B92] Tsai J. L. Simeonova D. I. Watanabe J. T. (2004). Somatic and social: Chinese Americans talk about emotion. *Pers. Soc. Psychol. Bull.* 30 1226–1238. 10.1177/0146167204264014 15359024

[B93] Türker E. (2013). A Corpus based approach to emotion metaphors in Korean: a case study of anger, happiness, and sadness. *Rev. Cogn. Linguist.* 11 73–144. 10.1075/rcl.11.1.03tur

[B94] White G. M. (1982). The role of cultural explanations in somatization and psychologization. *Soc. Sci. Med.* 16 1519–1530. 10.1016/0277-9536(82)90067-3 7135026

[B95] Wierzbicka A. (1992). Talking about emotions: semantics, culture, and cognition. *Cogn. Emot.* 6 285–319. 10.1080/02699939208411073

[B96] Wierzbicka A. (1995). The relevance of language to the study of emotions. *Psychol. Inq.* 6 248–252. 10.1207/s15327965pli0603_13

[B97] Wierzbicka A. (1999). *Emotions across Languages and Cultures: Diversity and Universals.* Cambridge: Cambridge University Press.

[B98] Wu D. Y. H. Tseng W. S. (1985). “Introduction: the characteristics of chinese culture,” in *Chinese Culture and Mental Health*, eds Tseng W. S. Wu D. Y. H. (Orlando, FL: Academic Press), 3–13.

[B99] Wu S. Keysar B. (2007). The effect of culture on perspective taking. *Psychol. Sci.* 18 600–606. 10.1111/j.1467-9280.2007.01946.x 17614868

[B100] Yamagishi T. Hashimoto H. Schug J. (2008). Preferences versus strategies as explanations for culture-specific behavior. *Psychol. Sci.* 19 579–585. 10.1111/j.1467-9280.2008.02126.x 18578848

[B101] Yamagishi T. Suzuki N. (2009). “An institutional approach to culture,” in *Evolution, Culture, and the Human Mind*, eds Schaller M. Norenzayan A. Heine S. J. Yamagishi T. Kameda T. (New York, NY: Psychology Press), 185–203.

[B102] Zane N. W. S. Yeh M. (2002). “The use of culturally-based variables in assessment: studies on loss of face,” in *Asian American Mental Health: Assessment Theories and Methods*, eds Kurasaki K. S. Okazaki S. (New York, NY: Kluwer Academic/Plenum Press), 123–138.

[B103] Zech E. (2000). *The Effects of the Communication of Emotional Experiences*. Doctoral dissertation, University of Louvain, Belgium.

[B104] Zhou X. Dere J. Zhu X. Yao S. Chentsova-Dutton Y. E. Ryder A. G. (2011). Anxiety symptom presentations in Han Chinese and Euro-Canadian outpatients: is distress always somatized in China? *J. Affect. Disord.* 135 111–114. 10.1016/j.jad.2011.06.049 21794924

